# Analysis of the A-U Rich Hairpin from the Intergenic Region of Tospovirus S RNA as Target and Inducer of RNA Silencing

**DOI:** 10.1371/journal.pone.0106027

**Published:** 2014-09-30

**Authors:** Marcio Hedil, Afshin Hassani-Mehraban, Dick Lohuis, Richard Kormelink

**Affiliations:** Laboratory of Virology, Department of Plant Sciences, Wageningen University, Wageningen, the Netherlands; University of British Columbia, Canada

## Abstract

Earlier work indicated that *Tomato spotted wilt virus* (TSWV) messenger transcripts, and not the (anti)genomic RNAs, are targeted by the RNA silencing machinery. Here, the predicted AU-rich hairpin (HP) structure encoded by the intergenic region (IGR) of the TSWV S RNA, and present at the 3′ end of viral mRNAs, was analyzed as a target and inducer for RNA silencing. Virus-derived siRNAs (vsiRNAs) purified from virus infected plants were found to derive from all three genomic RNA segments but predominantly the ambisense M and S RNAs. Further profiling on the S RNA sequence revealed that vsiRNAs were found from almost the entire S RNA sequence, except the IGR from where hardly any vsiRNAs were found. Similar profiles were observed with the distantly related *Tomato yellow ring* tospovirus (TYRV). Dicer cleavage assays using *Drosophila melanogaster* (*Dm*) embryo extracts showed that synthetic transcripts of the IGR-HP region were recognized as substrate for Dicer. Transient agroinfiltration assays of a GFP-sensor construct containing the IGR-HP sequence at its 3′ UTR (GFP-HP) did not show more rapid/strong silencing and profiling of the corresponding siRNAs, generated outside the context of a viral infection, still revealed relatively low levels of IGR-HP-derived siRNAs. These data support the idea that the IGR-HP is a weak inducer of RNA silencing and only plays a minor role in the amplification of a strong antiviral RNAi response.

## Introduction

RNA silencing, also named post transcriptional gene silencing (PTGS), is a conserved cellular mechanism in plants and animals in which double-stranded (ds)RNA, imperfect hairpin RNAs or highly structured single-stranded (ss)RNA trigger a chain of processes leading to sequence-specific RNA degradation [1], [2]. During this process, dsRNA is processed into small interfering RNAs (siRNAs) or microRNAs (miRNAs) of 21–26 nucleotides in length by RNase-III-type enzymes called Dicer or dicer-like (DCL) [3]–[7]. One strand of the siRNA duplex, named guide strand, is incorporated into the RNA-induced silencing complex (RISC) based on thermodynamic stabilities at the two ends [8], [9]. The RISC complex, being activated with the guide strand and a member of the Argonaute (Ago) protein family, continuously mediates recognition and subsequent cleavage of (m)RNA target sequences with complementarity to the siRNA guide strand, leading to endogenous or transgene silencing [10]–[12].

Plant viruses also induce RNA silencing often referred to as Virus-Induced Gene Silencing (VIGS), as can be observed by the generation of viral specific siRNA molecules during the infection process [13]. To escape from this antiviral defence mechanism, viruses have developed ways to counteract or evade it. One way that has been postulated for viruses to evade from RNA silencing is by inducing membrane cavities to replicate in (e.g. *Brome Mosaic virus*) and thereby avoiding exposure of viral dsRNA molecules to dicer [14]. Many plant viruses, though, encode proteins that are able to suppress RNA silencing by direct interference in the cascade of reactions that eventually leads to viral RNA degradation. Some RNA silencing suppressors (RSS) have been shown to inhibit silencing by sequestering siRNAs (NS3, NSs, P19) thus preventing their incorporation into RISC, whereas others avoid cleavage of dsRNA into siRNAs (HC-Pro), systemic transport of siRNAs (2b) or combinations of these [15]–[22]. In some other cases, the RSS protein interferes with protein components of the RNAi pathway (e.g. at the level of AGO1, DCL and RDR), and prevent maturation of the RISC complex or cleavage of RNA target sequences [13], [15], [23], [24]. In all of these cases, the final outcome is similar, i.e. viral RNA target molecules are prevented from becoming degraded by the RISC complex.

In contrast to the increasing insight into the working mechanisms of plant viral suppressor proteins, information on the origin of dsRNA molecules that induce VIGS still remains limited for many viruses. For RNA viruses it is generally assumed that ds replicative intermediates play a role in this, but nice examples exist, e.g. from *Cymbidium ring spot tombusvirus* [2], [25], in which cloning and sequence analysis of siRNAs from virus infected plants have revealed more siRNAs from the (+) strand than the (−) strand, pointing towards regions within the genomic RNA and intramolecular hairpin structures as a source of dsRNA for the production of siRNAs.

In plants silencing requires an amplification step involving a host RNA-dependent RNA polymerase (RDR) and this may occur in two ways. In the first way, primary siRNAs recruit RDR to homologous RNA molecules that serve as template for the generation of complementary RNA, thereby generating dsRNA from which secondary siRNAs are synthesised. In the second way, aberrant RNA molecules that arise as incomplete viral transcripts or resulting from RISC-mediated RNA target cleavage are recognised by RDR independent from primary siRNAs, and used as template to generate dsRNA. The amplification not only results in the production of secondary siRNAs identical to the dsRNA inducer sequence but also to the adjacent regions of target mRNA. This phenomenon of silencing spreading along the entire mRNA target sequence is referred to as transitive RNA silencing [26].

**Figure 1:**
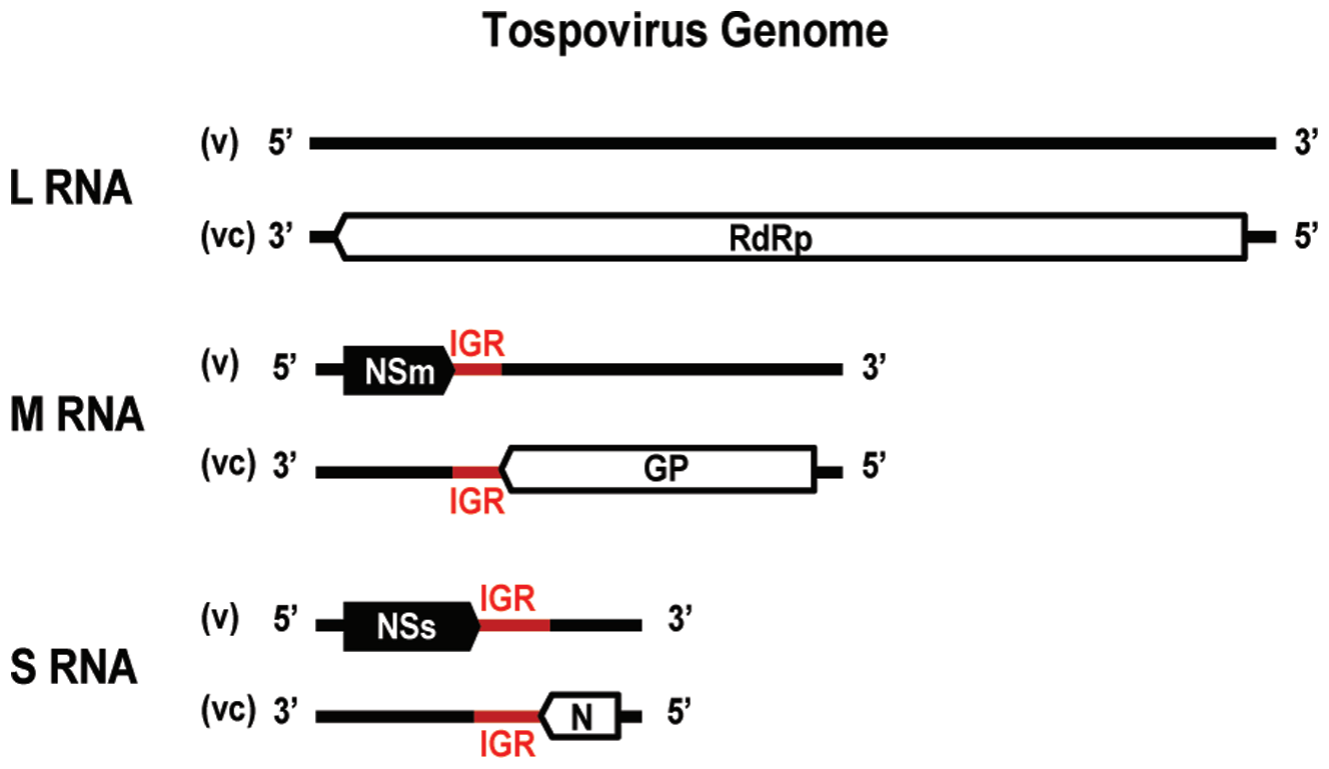
Schematic representation of the tospovirus tripartite RNA genome.

Tospoviruses, with *Tomato spotted wilt virus* (TSWV) as its representative, are the plant-infecting members of the arthropod-borne *Bunyaviridae*, a family that primarily consists of animal infecting viruses [27], [28]. Tospoviruses have a tripartite single-stranded RNA genome of negative/ambisense polarity. The segments are denoted, according to their sizes, as large (L), medium (M) and small (S) (Fig. 1). The viral (v) L RNA segment is of negative polarity and encodes the viral RNA-dependent RNA-polymerase (vRdRp) in the viral complementary RNA strand [29]. Both M and S RNA segments are of ambisense polarity and their genes are expressed via the synthesis of subgenomic messenger RNAs (sg-mRNAs) [30]. The M RNA segment encodes the precursor of the two glycoproteins Gn and Gc in the viral-complementary (vc) RNA strand and, in the viral (v) RNA strand, the putative cell-to-cell movement protein (NSm) [31], [32]. The S RNA segment encodes the nucleoprotein (N) in the vcRNA and the tospoviral suppressor of RNA silencing (NSs) in the vRNA [33]–[35].

**Figure 2:**
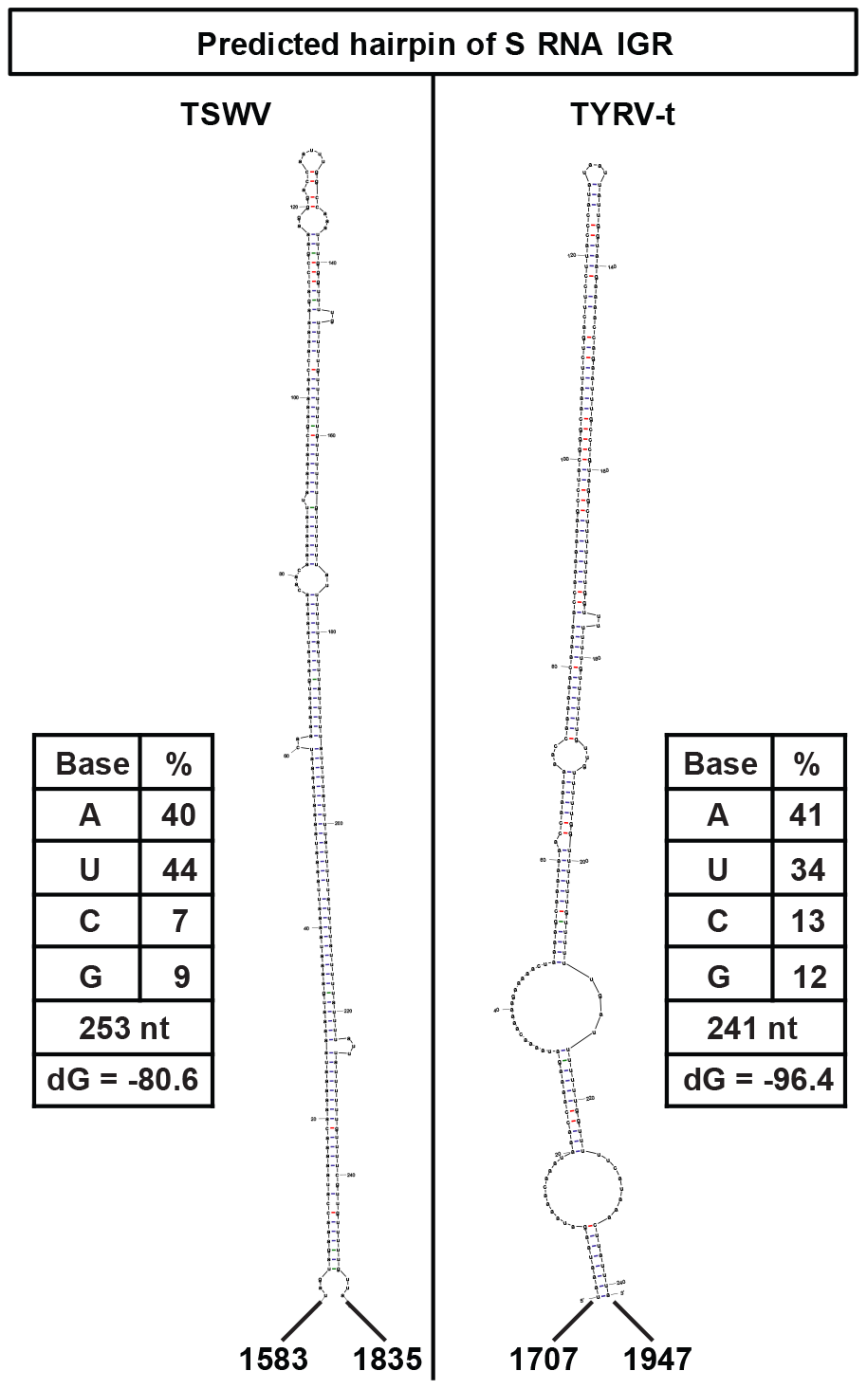
Folding prediction of A-U rich hairpin structures from tospovirus S RNA IGR: TSWV (left panel) and TYRV (right panel).

Ambisense RNA segments are relatively unique and besides tospoviruses, only found with members of the family *Arenaviridae*, the floating genus *Tenuivirus* and the genus *Phlebovirus* within the *Bunyaviridae* [36]. They are characterized by the presence of two non-overlapping open reading frames (ORFs) on opposite strands and separated by an intergenic region (IGR) of a few hundred nucleotides. Genes from ambisense RNA segments are generally expressed by the synthesis of sub-genomic length messenger RNAs that terminate in the IGR. The TSWV ambisense S and M RNA encoded IGRs are highly rich in A- and U- stretches and predicted to fold into a stable hairpin structure (HP) (Fig. 2) [31], [33]. Upon their formation, these are proposed to act as a transcription termination signal. This is supported by transcription studies, that have mapped the site of transcription termination of both TSWV S RNA encoded genes (N and NSs) to the 3′ end of the IGR [37], indicating that viral transcripts of the S RNA contain the predicted HP at their 3′ ends.

Considering the presence of long stretches (30–40 nts) of almost full complementarity within the predicted IGR encoding HP, and thus within viral mRNA transcripts, here the TSWV S RNA-derived IGR-HP was investigated as a potential target and inducer of RNA silencing *in planta*. To confirm that the findings where likely generic to all tospoviruses, the S-RNA-derived IGR-HP from *tomato yellow ring virus* (TYRV), another distinct (Asian) tospovirus, was included in the analysis. Results demonstrate that synthetic IGR-HP transcripts are recognized as dsRNA substrate during dicer-cleavage assays but during tospovirus infection, as well as during transient expression in the absence of NSs, hardly any siRNAs are produced from the IGR-HP.

## Results

### TSWV and TYRV infections predominantly lead to production of M- and S RNA-specific vsiRNAs

**Figure 3:**
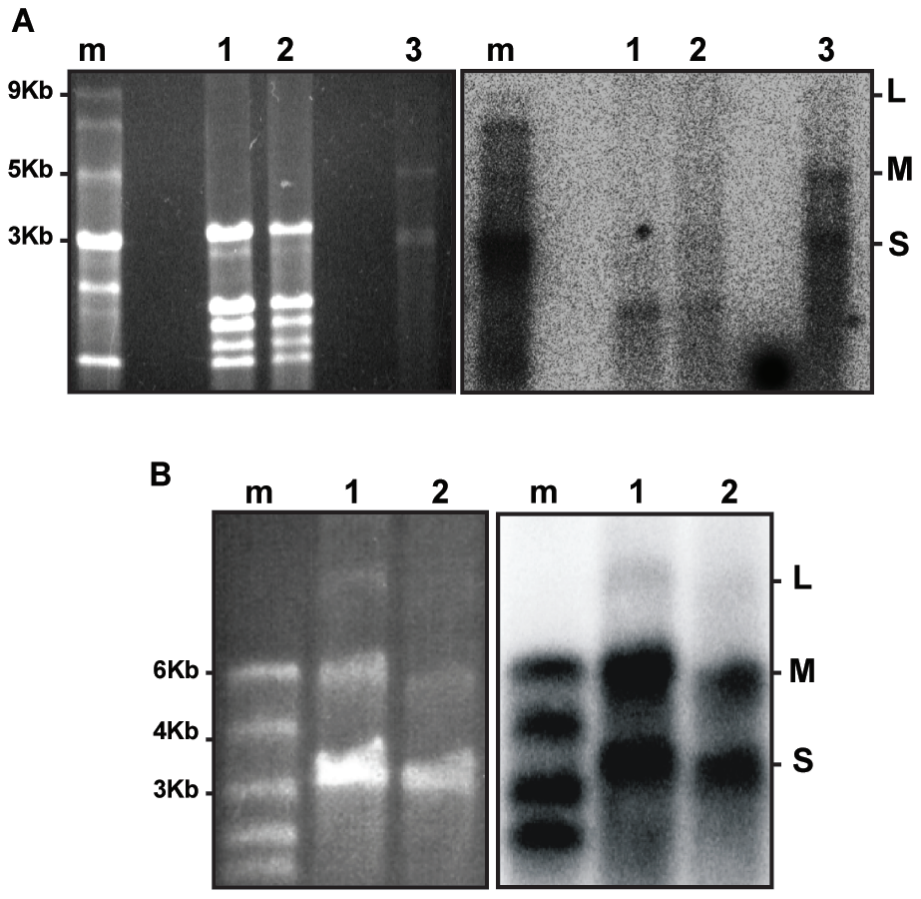
Production of vsiRNAs from tospoviral S, M and L genomic RNA segments. (A) Total RNA from healthy (lane 1) and TSWV infected *N.benthamiana* (lane 2); genomic RNA from TSWV RNPs (lane 3). As a size marker (m), ssRNA Ladder (NEB) was used. (B) Genomic RNA from TYRV RNPs, undiluted (lane 1) and diluted 1x (lane 2). As a size marker (m), RiboRuler High Range RNA Ladder (Thermo Scientific) was used. Left panel presents agarose gel. Right panel presents Northern blot hybridized with radiolabeled siRNAs purified from TSWV or TYRV-infected *N. benthamiana*.

A common feature to all tospoviruses is the presence of an IGR within the ambisense M and S RNA segments, that contains long stretches of A-rich and U-rich sequences and is predicted to fold into a stable HP (Fig. 2). Based on the presence of these structures, it is tempting to hypothesize that the presence of these in viral mRNA turns them into potent inducers (and targets) of antiviral RNAi. If this is true, more vsiRNAs are expected to correspond to the ambisense M and S RNA segments in comparison to the L RNA segment that lacks such IGR sequence. To test for this, and analyse whether M and S RNA indeed give rise to the production of higher levels of vsiRNAs, small RNA molecules were purified from TSWV-infected *N. benthamiana* leaf material and, after radiolabeling, probed on total RNA and genomic RNA purified from isolated viral RNPs (Fig. 3A).

While vsiRNAs were found hybridizing to the L, M and S RNA segments, strong hybridization signals were observed with the ambisense M and S RNA segments (Fig. 3A, lane 3). Hybridization signals on total RNA purified from TSWV infected leafs were weak, likely due to the relative lower amounts of viral RNA in these fractions (Fig. 3A, lane 2). To test whether this pattern of vsiRNAs was common to other tospoviruses, the same experiment was performed with another distinct tospovirus, *Tomato yellow ring virus* (TYRV) [38], from which the S RNA IGR was earlier observed to contain extensive stretches of full complementarity (Fig. 2B). The results again revealed the generation of relatively high amounts of vsiRNAs derived from the M and S segments and only low amounts from the L RNA (Fig. 3B, lanes 1 and 2).

### Non-uniform production of vsiRNAs along the tospovirus S RNA sequence

**Figure 4:**
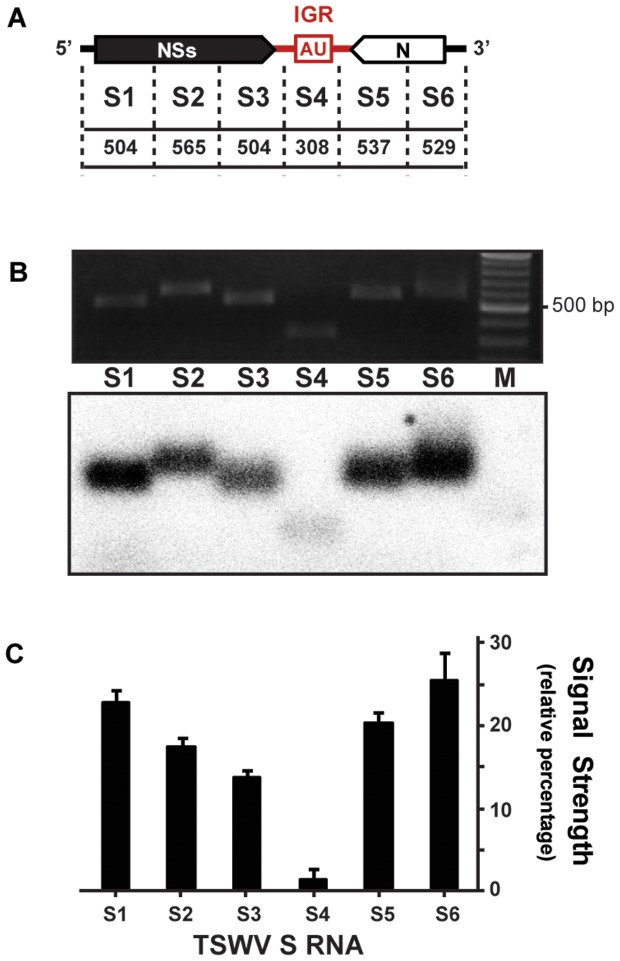
Distribution of vsiRNAs on TSWV ambisense S RNA segment. (A) Schematic representation of TSWV S RNA segment. Intergenic region (IGR), with predicted hairpin structure (AU box), is indicated in red. PCR fragments spanning S RNA (S1 to S6) respective basepair sizes are indicated; dotted lines roughly demark positions of primers used. (B) Ethidium bromide staining of agarose gel containing fragments S1 to S6 (upper panel), and corresponding Southern blot hybridized to radiolabeled siRNAs purified from TSWV-infected *N. benthamiana* (lower panel). (C) Relative signal strength of siRNAs on each genomic cDNA fragment. Standard error of mean (SEM) from two independent experiments is indicated.

To test whether the vsiRNAs originating from the ambisense M and S RNA segments predominantly corresponded to the IGR encoded HP, suggestive for the status of HP as strong inducer/target of RNA silencing, the vsiRNAs were further fine mapped on the S RNA segment. To this end, radiolabeled TSWV vsiRNAs were hybridized to similarly sized PCR fragments spanning the entire S RNA segment. Although vsiRNAs hybridized to sequences covering the entire TSWV S RNA segment, and good amounts were obtained from sequences of the NSs and N genes (Fig. 4A and 4B), unexpectedly, hardly any siRNAs originated from the IGR encoded HP sequence (Fig. 4B and 4C). No signals were observed when small RNAs purified from healthy plants were used as probe (data not shown).

**Figure 5:**
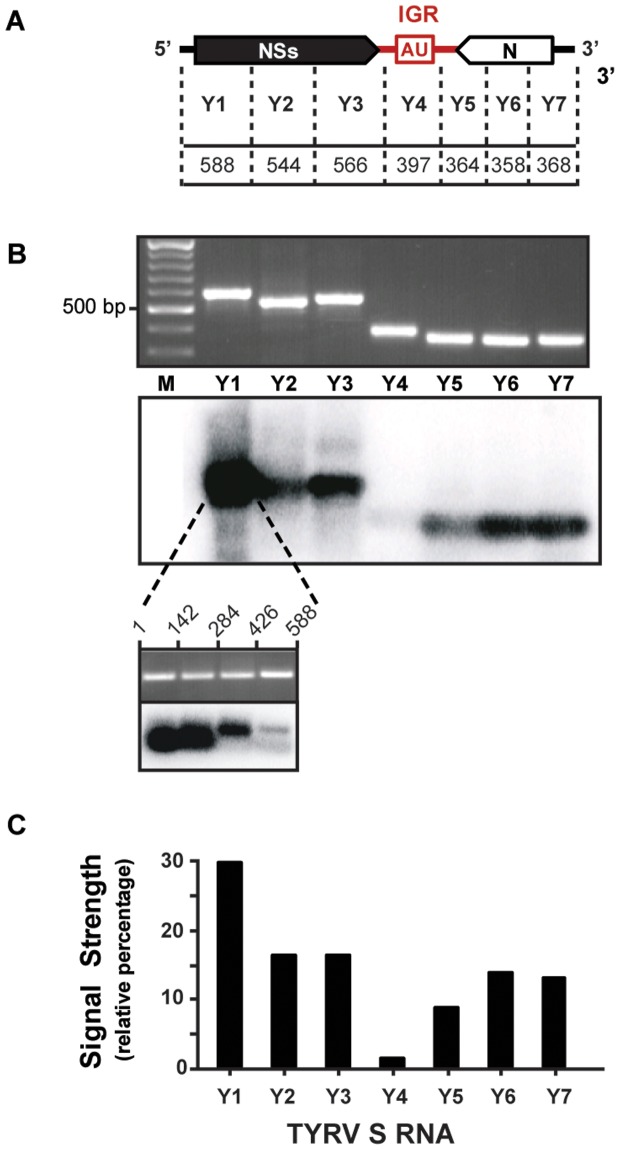
Distribution of vsiRNAs on TYRV ambisense S RNA segment. (A) Schematic representation of TYRV S RNA segment. Intergenic region (IGR), with predicted hairpin structure (AU box), is indicated in red. PCR fragments spanning S RNA (Y1 to Y7) respective basepair sizes are indicated; dotted lines roughly demark positions of primers used. (B) Ethidium bromide staining of agarose gel containing PCR fragments Y1 to Y7 (upper panel), and corresponding Southern blot hybridized to radiolabeled siRNAs purified from TYRV-infected *N. benthamiana* (lower panel). Below, fine mapping of fragment Y1. (C) Relative signal strength of siRNAs on each genomic cDNA fragment.

To verify whether a similar vsiRNA distribution profile would be obtained with TYRV, a similar fine mapping study was performed for this virus. Like TSWV, TYRV infections gave rise to S RNA-derived vsiRNAs that mapped to all regions of the S RNA segment (Fig. 5A and B), but those from the IGR encoded HP structure were relatively scarce (Fig. 5B and C). Furthermore, almost twice as much vsiRNAs were observed to originate from the start region of the NSs ORF (fragment Y1; position 1-588 in the vRNA), when compared to other regions of the S RNA (Fig. 5B and 5C). A further fine mapping within this region revealed that siRNAs specifically derived from the nucleotide sequence 1-284 from TYRV S RNA (Fig. 5B, lower panel). No signals were observed when siRNAs purified from healthy plants were used as probe (data not shown).

### HP transcript is cleaved by Dicer *in vitro*

**Figure 6:**
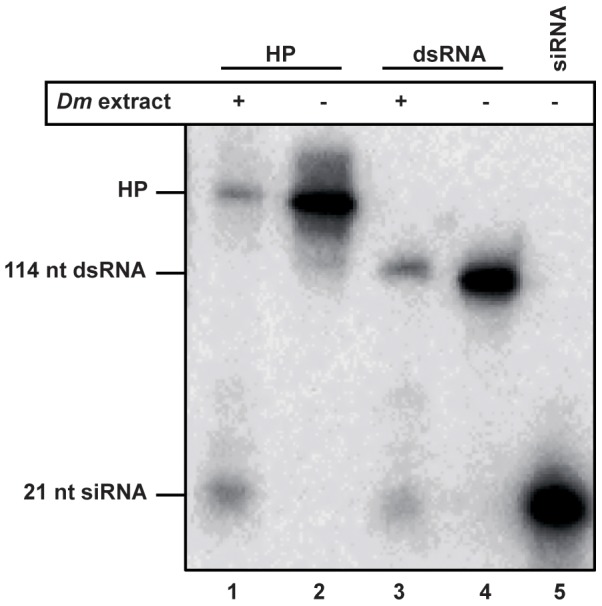
Dicer-mediated cleavage of hairpin transcripts (HP) from TSWV S RNA IGR-encoded hairpin sequence. Radioactively labeled HP transcripts (lane 2) were incubated in the presence of dicer containing *Drosophila melanogaster* (*Dm*) embryo extracts and cleavage products (lane 1) subsequently resolved on 8% denaturing acrylamide gel. As positive control, 114-nt dsRNA (lane 4) was included to verify dicer activity from *Dm* extracts (lane 3). As size marker, radiolabeled 21nt siRNAs were included (lane 5).

While only few vsiRNAs were found mapping to the IGR encoded predicted hairpin-structure, this region was further investigated as potential inducer and target of antiviral RNAi in a dicer cleavage assay. To this end, synthetic radiolabeled transcripts of the TSWV IGR-encoding HP sequence were made and after being allowed to fold into a dsRNA hairpin structure, subsequently offered to RNAi-induced *Drosophila melanogaster* (*Dm*) embryo extracts containing Dicer-1 and Dicer-2 [6], [39]. Analysis of the products on non-denaturing acrylamide gels showed that the HP transcript was cleaved into small RNAs, co-migrating with siRNAs (21 nucleotides) cleaved from a 114 nt dsRNA transcript and with the siRNA size marker (Fig. 6). Similar results were obtained when using synthetic transcripts from the TYRV S RNA IGR sequence (data not shown) and support the idea that the IGR encoding hairpin structure is recognized as a substrate for dicer.

### The IGR-encoded HP-structure sequence is weakly targeted by the RNAi machinery during transient expression *in planta*

**Figure 7:**
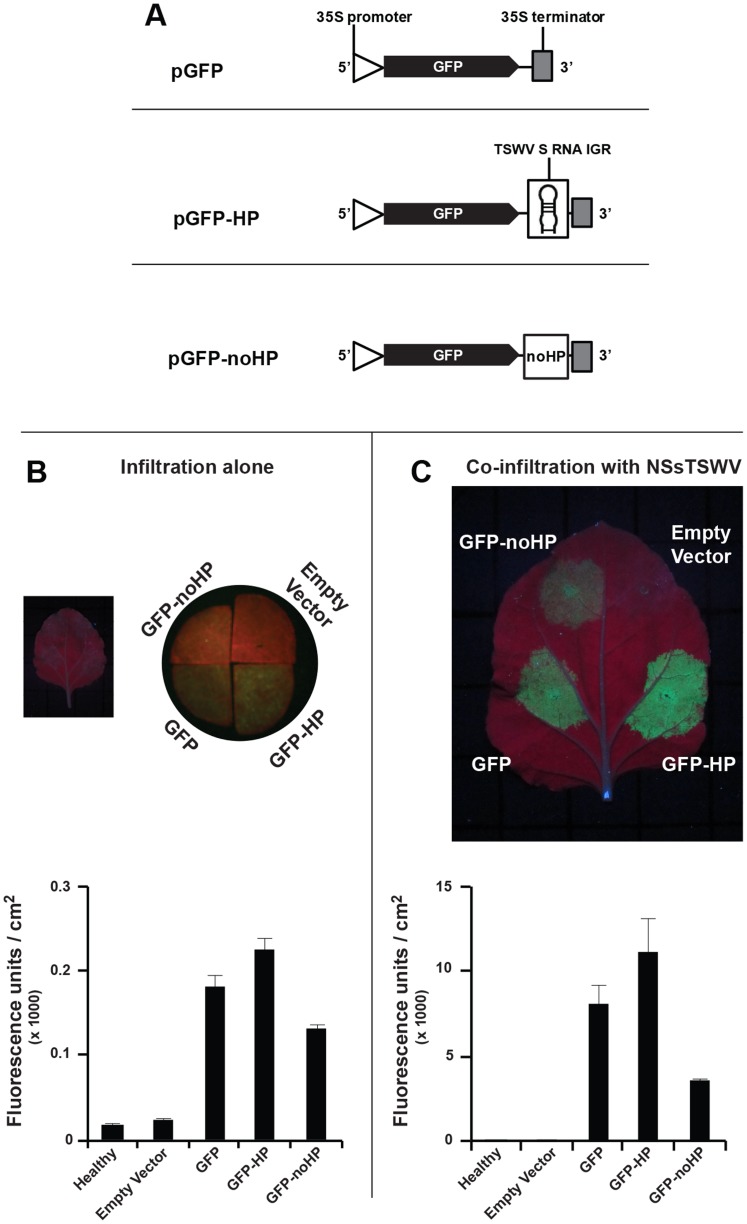
Agroinfiltration leaf patch assays of GFP gene constructs containing 3′ hairpin trailer. (A) Schematic representation of GFP constructs containing the different 3′ trailer sequences analyzed. The noHP sequence consists of a partial N gene sequence in antisense polarity. (B) Transient GFP expression after agroinfiltration of GFP constructs in absence of RSS. As only very low levels of fluorescence were visual at first (left), leaf disks were further analysed on binocular stereomicroscope M3Z, Leica (right). (C) Similar as panel B, but in the additional presence of TSWV NSs. Fluorescence in panels B and C was quantified and depicted in the graphs underneath. Standard error of mean (SEM) from three leaf disks is indicated.

While synthetic transcripts from the IGR encoded HP structure were recognized as substrate for dicer, the presence of only low amounts of vsiRNAs derived from this sequence during a natural infection could be due to the possibility that the hairpin structure is being protected from Dicer cleavage by a viral protein, e.g. the TSWV NSs RSS protein. If this is true, elevated levels of HP-derived siRNAs would be expected when the HP structure is expressed outside the context of a viral infection. To test this hypothesis, and further investigate the IGR HP structure as a potential target of RNAi, a functional GFP construct was made containing the TSWV HP structure sequence at its 3′ end (and denoted GFP-HP, Fig. 7A) and next expressed during an agroinfiltration leaf patch assay on *N*. *benthamiana*. As controls, GFP constructs were included that either lacked the entire HP-structure sequence (GFP) or contained part of an antisense N gene sequence that was shown to be well targeted by the silencing machinery during a natural virus infection and predicted to not fold into a stable hairpin structure (GFP-noHP, Fig. 7A). As expected, several days post agroinfiltration, GFP expression from the control construct became silenced but a comparative analysis of all constructs did not reveal a stronger silencing of GFP in the presence of a 3′ sequence for the predicted HP structure. Instead, and somewhat surprising, higher levels of GFP expression were consistently observed with GFP-HP during repeated experiments, and suggestive of a lower silencing, in the absence (Fig. 7B) or presence of the TSWV NSs RSS protein (Fig 7C), compared to the other GFP constructs. Silencing of GFP expressed from the construct GFP-noHP consistently appeared most strongest, and this was supported by the observation that in the additional presence of the NSs RSS protein, the levels of GFP were still lower compared to those from the GFP-HP and GFP constructs.

**Figure 8:**
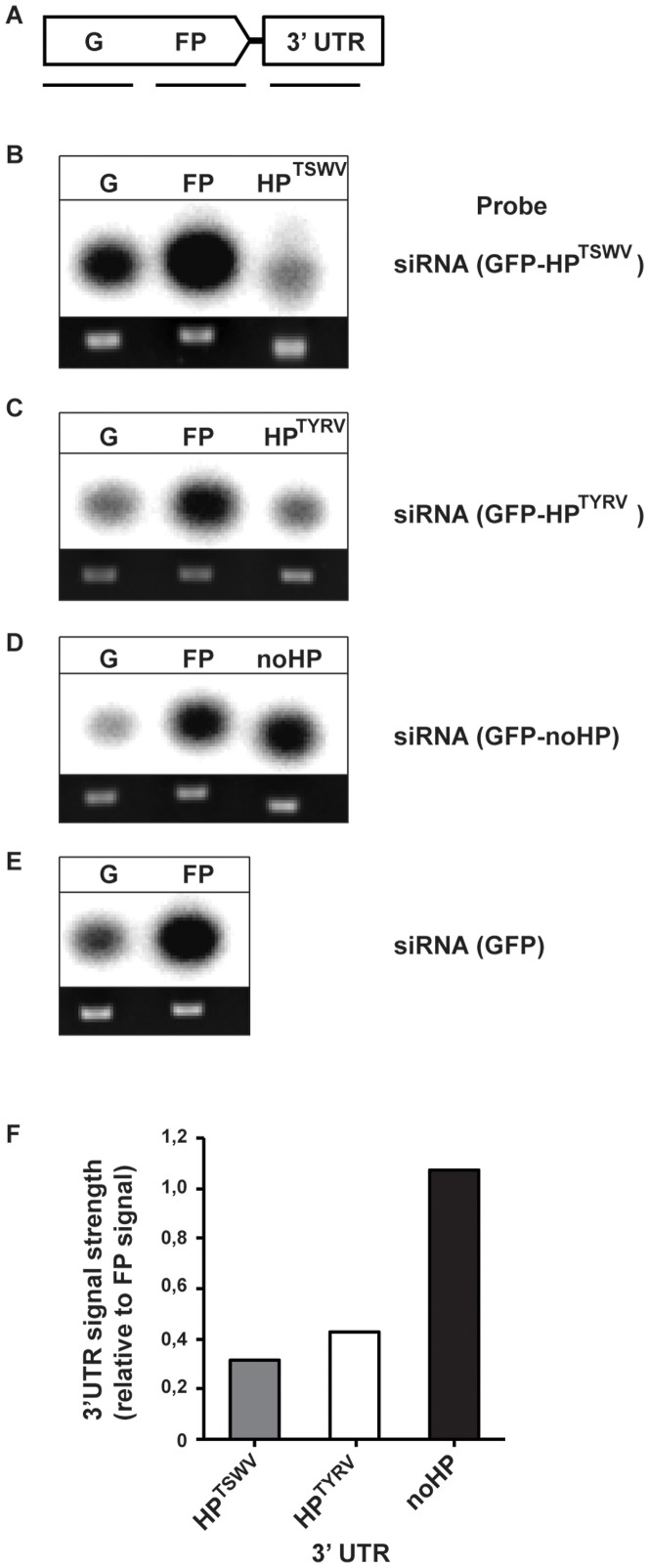
Production and distribution of siRNAs from GFP constructs containing various 3′ trailer sequences. Small RNAs purified from transient expression of GFP constructs were probed on Southern blots containing PCR fragments spanning the respective construct sequence. (A) Schematic view of constructs and PCR products spanning the sequence. The noHP sequence consists of a partial N gene sequence in antisense polarity. Southern blot analysis of constructs: (B) GFP-HP^TSWV^; (C) GFP-HP^TYRV^; (D) GFP-noHP; (E) GFP. Ethidium bromide-staining of PCR products are shown below. (F) Graphical representation of the siRNA signal strength corresponding to the 3′ trailer sequences and normalized to the signal strength of the 3′ half of GFP (FP) of each construct. Abbreviation: G: 5′ half of GFP; FP: 3′ half of GFP; HP: A-U rich hairpin structure (from IGR of TSWV and TYRV S RNA); noHP: part of TSWV N gene.

To investigate whether in the absence of viral proteins, the HP structure *in planta* was more targeted by RNAi and lead to relatively enhanced siRNA levels, fractions of small RNA were purified from the leaf tissues collected from the agroinfiltration leaf patch assays and probed on PCR fragments presenting the 5′ half (denoted “G”, Fig. 8A) or 3′ half (“FP”, Fig. 8A) of GFP sequence or the added IGR-encoding HP structure sequence. Analysis of the results showed a consistent production of similar and high amounts of siRNAs originating from the 3′ half of the GFP gene (FP) compared to its 5′ half (G) for all GFP gene constructs, regardless of the presence or absence of a 3′ trailer sequence in the construct (Fig. 8B-E). On the other hand, still relatively few siRNAs were observed to derive from the IGR encoded HP sequence of TSWV within the GFP-HP^TSWV^ construct (Fig. 8B), similar to the situation of a natural viral infection (Fig. 4B and C, lanes S3 and S4). Furthermore, siRNAs originating from the added 3′ trailer sequence within the control construct GFP-noHP were produced in high and similar amounts relatively to siRNAs originating from the 3′ half of the GFP sequence (FP) (Fig. 8D). Since GFP-noHP was silenced most strongly during agroinfiltration leaf patch assays, and only differed from the other constructs in the 3′UTR, this indicated that its 3′UTR presented a stronger target for RNAi compared to the one from GFP-HP, and relative to the siRNA signals from the internal 3′ half of the GFP gene (FP) (Fig. 7B and C). Results similar to those for GFP-HP^TSWV^ were observed when the HP of TYRV was added as a trailer sequence to GFP (GFP-HP^TYRV^, Fig. 8C). This was supported by quantifying the siRNA-signal strength of the 3′ trailer sequences normalized to the signal strength of the 3′ half of GFP (FP) for each construct (Fig. 8F). Altogether, these data indicate that even in the absence of viral proteins the HP structures of TSWV and TYRV S RNA are weak targets/inducers of RNA silencing. No signals were observed using small RNAs purified from healthy leafs or agroinfiltrated with an empty binary vector, or when probing 3′trailer sequences with small RNAs purified from leafs agroinfiltrated with the GFP control construct (data not shown).

## Discussion

RNA silencing, besides being involved in host gene regulation and developmental processes, is an antiviral defence mechanism induced by dsRNA and imperfect hairpin RNAs. Here evidence is presented indicating that the predicted HP structure sequence encoded by the IGRs of TSWV and TYRV S RNA, is a suitable target for DCR1 and DCR2 from *Drosophila* extracts [6], but only plays a minor role in the induction/amplification of a strong antiviral RNAi response.

Tospoviral RNA genome segments are known to be tightly encapsidated with N protein and therefore not freely exposed to become targeted by RNA silencing, in contrast to their (sub)genomic mRNA molecules [30]. The latter is supported by the observation that TSWV is still able to replicate in protoplasts from TSWV NSm transgenic plants that confer RNAi-mediated resistance to TSWV [40]. The siRNAs produced and corresponding to N and NSs ORFs (Fig. 4 and 5) thus most likely result from silencing of their corresponding messenger transcripts and not from the genomic S RNA segment. The same explanation likely holds for siRNAs derived from the M and L RNA (Fig. 3).

Considering that the ambisense encoded tospoviral N and NSs transcripts contain a 3′ UTR consisting of the IGR-encoding HP structure sequence [37], instead of a regular eukaryotic poly(A)-tail, they were speculated to present a perfect target and inducer of antiviral RNAi. Surprisingly, the IGR-encoding HP structure sequence only gave rise to very small amounts of siRNAs during a natural infection, as also observed in a recent deep sequencing study analysis on TSWV infected plants [41]. On the other hand, dicer cleavage assays showed that the IGR-encoded HP structure sequence does present a suitable target for Dicer (Fig. 6), indicating that this structure is likely masked during a natural infection cycle. Whether the predicted HP structure (Fig. 2) during *in vitro* dicer cleavage assays is recognized as dsRNA or as an imperfect hairpin RNA somewhat resembling precursors to miRNAs is not clear yet, since *Drosophila* embryo extracts contain both DCR1 and DCR2, of which DCR1 is normally resident to the nucleus and involved in miRNA production whereas DCR2 localizes to the cytoplasm and produces siRNA [6].

The idea of the predicted HP structure-sequence being protected from cleavage by DCL *in planta* is strengthened by the observation that transient expression of a GFP construct containing a 3′ IGR-HP structure sequence did not reveal an elevated level of HP-derived siRNAs either and, relative to the 3′ part of the preceding ORF (Fig. 8, part FP of the GFP gene), showed similarity to the siRNA level produced from this sequence during a natural infection cycle, while the amounts from the N gene-based 3′UTR control sequence were relatively equal to those from the 3′ part of the preceding ORF. The latter clearly indicated that the N gene based 3′UTR sequence was similarly accessed for siRNA-processing as its upstream sequence, whereas the HP sequence somehow remained protected from this, even outside the viral context. How the IGR-HP is being protected from recognition by the RNAi silencing machinery remains to be further investigated. However, an earlier study showed that translation of luciferase gene constructs was supported in the presence of various 3′ trailer sequences consisting of the tospoviral HP, and this even became enhanced in the additional presence of NSs [42], which indicated that the IGR-HP could act as a functional equivalence of a poly(A)-tail. Together with the results showing that the HP sequence, even outside a viral context, is only being processed into siRNAs to a limited extent makes it tempting to speculate that the IGR-HP structure is masked from the RNA silencing machinery by proteins involved in the translational machinery. As suggested earlier [41] and in light of the A-rich part of the IGR-HP, the cellular PABP could present a candidate for this. During a natural infection this may involve the additional action of the tospoviral NSs protein, considering that it has been shown to be able to bind long dsRNA [16], and thereby support its earlier observed enhancement of translation effect on mRNAs containing a 3′ IGR-HP [42]. According to this idea, the IGR-HP structure sequence would then be engaged most of the times in viral/host protein interactions and inaccessible for siRNA generation by RNase-III type enzymes or to assist in the generation of secondary siRNAs by RDR. In light of the structural similarities, this would not only apply to the S RNA, but also to the ambisense M RNA encoded transcripts where similar, stable hairpin structures are predicted [31].

Our observations on siRNAs from the IGR-encoded HP structure sequence are supported by recent deep sequencing data [41], [43], however in both studies the relative lower amounts of vsiRNAs produced from the S and M RNA encoded IGR sequences were not remarked by the authors.

The observations of high amounts of siRNAs mapping to the NSs gene is interesting in light of this protein acting as a suppressor of silencing [34], [35] and when considering the RNA silencing effect on viral replication and plant-virus dynamics [44]. Folding predictions of the RNA sequence around the start of the TYRV NSs ORF revealed a small hairpin structure (NSs-hairpin), and similar ones at almost the same position were found in several other tospoviruses. Hence, though speculative, the presence of an RNAi target within the NSs gene might be involved in regulating NSs expression and, consequently, tospovirus virulence.

In conclusion, the AU-rich hairpin structure in the tospoviral IGR presents a suitable substrate for Dicer but appears to present only a weak inducer and target of RNAi, likely due to being masked by viral and/or host proteins. Elucidating the nature of these will provide further insight into the role of the hairpin structure in processes of viral transcription and translation.

## Materials and Methods

### Viruses and Plants

The tospovirus strains TSWV BR-01 [45] and *Tomato yellow ring virus*-tomato strain (TYRV-t, here referred simply as TYRV) [38] were maintained by mechanical passage on hosts *Nicotiana benthamiana* and *N. rustica* cv. America.

### Detection, isolation and labeling of siRNAs from plant leaves

Isolation of small RNAs was performed as previously described [46], [47]. In brief, leaf material (from healthy and systemically infected *N.benthamiana* leaves) was ground in liquid nitrogen and next mixed with extraction buffer (2% Sarcosyl – 5 M NaCl), followed by phenol extraction. The aqueous phase was collected and subjected to polyethylene glycol (PEG) precipitation [5], in order to separate low-molecular-weight (LMW) RNA molecules from DNA and larger RNA molecules. For the purification of siRNAs, 15 to 30 µg of LMW RNAs were resolved on a 15% denaturing polyacrylamide gel containing 8 M urea. After ethidium bromide staining, the region containing siRNAs was excised from the gel, ground to small pieces and incubated in 3 M NaCl overnight at 4°C to extract the siRNAs from the gel by diffusion. After centrifugation, the supernatant was collected and the siRNAs were ethanol precipitated. Small interfering RNA molecules were dephosphorylated with alkaline phosphatase and subsequently end-labeled with [γ-^32^P]-ATP (Perkin Elmer) by T4 polynucleotide kinase (Promega) according to the manufacturer's instructions.

### Purification of tospovirus genomic RNA from ribonucleoproteins (RNPs) and northern blotting

Tospoviral RNPs were purified from *N. rustica* cv. America as previously described [48]. Genomic RNA was purified using hot phenol extraction followed by ethanol precipitation [30]. Purified RNA was resolved in 1% agarose gel under RNase free conditions and blotted to Hybond-N membrane (Amersham Biosciences) by top-down blotting in neutral transfer conditions using Whatman TurboBlotter system according to manufacturer's instruction. Filters were hybridized to [γ-^32^P]-labelled siRNAs (see below) purified from healthy and tospovirus-infected *N.benthamiana* leaves.

### Southern blotting, siRNA purification and mapping on TSWV and TYRV S RNA

Total RNA was purified from systemically infected *N.benthamiana* leafs using Trizol (Life Technologies). The S RNA segment was RT-PCR-amplified, using Superscript RT (Invitrogen), in 6-7 fragments of similar size and spanning the entire S RNA segment from TSWV and TYRV respectively. The products were further cloned in pGem-T Easy (Promega) according to the manufacturer's instructions and verified by sequence analysis. For TYRV S RNA-specific fragments, equimolar amounts of PCR products were resolved on 1% agarose gel. For TSWV S RNA, due to difficulties in obtaining single PCR products, S RNA-specific fragments were excised from pGem-T Easy plasmid DNA and equimolar amounts resolved on 1% agarose gel. DNA was blotted to Hybond-N membrane (Amersham Biosciences) by top-down blotting. Filters were subsequently hybridized (at 48°C) overnight in Church buffer [49] to [γ-^32^P]-labelled siRNAs purified from healthy or tospovirus-infected *N.benthamiana* leaves. After washing, filters were exposed for two days to phosphor screen (Kodak) and visualized by phosphorimaging (Molecular Imager FX, Bio-Rad). Signal quantification was performed with ImageJ software [50].

### Synthesis of [^32^P]-radiolabelled dsRNA substrates

DNA templates of the A-U rich predicted hairpin encoding sequence (from TSWV S RNA IGR) (Fig. 2) were RT-PCR amplified using primers containing the T7 RNA polymerase promoter sequence. PCR fragments were purified using High Pure PCR purification kit (Roche) and radiolabelled RNA transcripts were prepared by *in vitro* transcription using T7 RNA polymerase (Promega) in the presence of [α-^32^P]-rNTP (PerkinElmer Inc., UK) according manufacture's instruction. Products from the *in vitro* transcription were resolved on an 8% denaturing acrylamide gel and the radiolabelled A-U rich predicted hairpin transcript was excised from the gel and extracted by diffusion into 20 µl 2x PK buffer (200 mM Tris pH 7.5, 300 mM NaCl, 5 mM EDTA, 2% SDS) followed by phenol chloroform and ethanol precipitation. Prior to use, purified RNA transcripts were briefly heated for 10 min. at 85°C and gradually cooled down to room temperature to allow RNA folding. 114-nt dsRNA molecules were prepared as previously described [16].

### Dicer cleavage assay (DCA)

*Drosophila melanogaster* (*Dm*) embryo extract was prepared as previously described [39]. In brief, for the dicer cleavage reactions a reaction mixture of 10 µl consisting of 5 µl *Drosophila* embryo extract, 5 nM ^32^P-labeled transcript of the IR hairpin or dsRNA were incubated for 2–3 h at 25°C [39], except potassium acetate was omitted from the reaction mixture [16]. Next, samples were deproteinized with proteinase K, RNA was phenol extracted and analyzed on 8% denaturing acrylamide gel, which were then dried for 30 minutes at 80°C, exposed to a phosphor screen (Kodak) for 12 hrs and scanned with PhosphorImager (Molecular Imager FX, Bio-Rad).

### *Agrobacterium tumefaciens* mediated transient expression assay (ATTA) of GFP-hairpin constructs *in planta*

To analyse the IGR hairpin as an inducer of silencing outside the context of a tospoviral infection, leaf patch assays with the *Agrobacterium tumefaciens* transient expression assay (ATTA) system were performed as previously described [35], [51]. To monitor the effect of the hairpin sequence on the induction of silencing of a functional green fluorescent protein (GFP) gene construct, the hairpin-encoding sequence (nucleotide position 1044–1368 and 1032–1427 of, respectively, TSWV and TYRV vc S RNA) was fused by PCR amplification to the 3′ end of the GFP gene, generating constructs GFP-HP^TSWV^ and GFP-HP^TYRV^. As a control, an inverted part of the TSWV N gene sequence (nucleotide position 235–528 of vc S RNA, corresponding to position 82–375 from ATG of N gene) was fused to the 3′ end of the GFP gene, resulting in the GFP-noHP construct. All GFP-HP, GFP-noHP and GFP constructs were cloned in binary vector pK2GW7 [52] using the Gateway Cloning Technology (Invitrogen). For suppression of silencing the TSWV *NSs* and tombusvirus *P19* genes were expressed from binary vectors pK2GW7 and pBin19, respectively. To this end, binary vectors were transformed to *Agrobacterium tumefaciens* strain cor308 [53] and cultured in LB3 medium containing appropriate antibiotics for selection (Tetracycline 2 µg/ml and Spectinomycin 250 µg/ml – for pK2GW7 – or Kanamycin 100 µg/ml – for pBin19) at 28°C overnight. From the overnight culture, 600 µl was transferred to 3 ml induction medium (10.5 g/l K_2_HPO_4_, 4.5 g/l KH_2_PO_4_, 1.0 g/l (NH_4_)_2_SO_4_, 0.5 g/l Sodium Citrate Dihydrate, 0.25 g/l MgSO_4_, 0.2% (w/v) glucose, 0.5% (v/v) glycerol, 50 mM acetosyringone and 10 mM MES pH 5.6) and grown at 28°C overnight. The induced culture was pelleted and ressuspended in Murashige-Skoog (MS) medium (30 g/l sucrose; 40 g/l MS; pH 5.7) containing 150 µM acetosyringone and 10 mM MES (pH 5.6) to an OD_600_ of 0.5. This suspension was used to infiltrate fully expanded leafs of *N*. *benthamiana* plants. Silencing of GFP and suppression by NS_S_ and P19 proteins was assessed by UV light and western blot analysis, respectively. To suppress silencing, RNA silencing suppressor constructs were provided in a co-ATTA with GFP constructs. To this end, induced *Agrobacterium* suspensions were mixed at a final OD_600_ of 0.5 prior to infiltration. Infiltrated plants were kept at 25°C and monitored for GFP fluorescence during a 5-day period using a GFP fluorescence-stereo-microscope. Pictures were taken at 5 dpi (days post infiltration). Quantification of GFP fluorescence from 1 cm^2^ leaf disk was performed using Fluorstar Optima (BMG Labtech) as previously described [54]. As probes for southern blotting, small RNAs were purified from 6 g of agroinfiltrated leafs and radiolabeled as described above.

### UV photography and quantification of GFP fluorescence

Pictures of whole leafs (as shown in Fig. 7B and 7C) were taken with a digital camera (Canon PowerShot A3200 IS) by using a hand-held UV light (Philips, 6W). In case of leafs agroinfiltrated with GFP constructs without a suppressor of RNA silencing, close-up UV pictures (as shown in Fig. 7A) were made using a digital camera CoolSnap and a binocular stereomicroscope (M3Z, Leica). For the quantification of GFP fluorescence, 5dpi leaf disks of 1 cm in diameter were taken from infiltrated leaf area and analysed using Fluorstar Optima (BMG Labtech), as previously described [54].

### Folding predictions for S RNA intergenic hairpin sequence

Folding predictions were performed at 37°C, using Mfold [55], [56].
